# Supplementary material for: Assessment of Anti-Bacterial Effectiveness of Hand Sanitizers Commonly Used in South Africa
Source: Int J Environ Res Public Health. 2022 Jul 28;19(15):9245. doi: 10.3390/ijerph19159245 (PMC9367797; doi:10.3390/ijerph19159245)
Supplement: Supplementary file 1 [file ijerph-19-09245-s001.zip › ijerph-1763094-supplementary.pdf]

Supplement S1: Label information of hand sanitisers selected.

Sample Identification	Active Ingredients	Kill Rate	SABS, SAHPRA approved	Application instructions
Hand sanitiser A	Chloroxedine, Gluconate and 70% alcohol	-	SABS only	Not indicated
Hand sanitiser B	70% ethanol, emollients, fragrance (parfum)	99.9%	No	Apply 2 ml on palm
Hand sanitiser C	Alcohol denat, water (aqua), triethanolamine, fragrance (parfum), carbomer, limonene, linalool, and 65% ethanol.	99.9%	No	Squeeze generous amount onto hands
Hand sanitiser D	Alcohol denat, carbomer, triethanolamine glycerine, Benzophenone, Sodium Orthophyl Phanate, aloe, ethylhexyglycerin		No	Apply onto hands volume not specified
Hand sanitiser E	Triethanolamine, Carbomer, Amy linabol.	99%	No	Squeeze generous amount
Hand sanitiser F	Alcohol denat, Propylene glycol, acrylates, alkyl, tetrahydroxypropyl, ethaylenedaimine chromomile.	99%	No	Thumbnail size amount
Hand sanitiser G	0.5 % Chlorohexidine and gluconate, 70% ethanol	-	SAHPRA only	2.5 ml -5 ml
Hand sanitiser H	Acrylates, Alkyl acrylate crosspolyer, glycerin honey, Isopropyl alcohol, ethyl alcohol 62% antimicrobial.	99.99 %	No	Wet Hands with sanitiser
Hand sanitiser I	Isopropanol 70 %	-	SABS only	Apply to hands
Hand sanitiser J	Alcohol, propylene glycol, isopropyl alcohol, Aminomethyl propanol, carbomer, tetrasodium.	99.9%	No	Apply on your palm
Hand sanitiser K	Alcohol denat, aqua, propylene glycol, glycereth-26, Acrylates/c10-30 alkyl, acrylate cross polymer, parfum, triethanolamine, citral, eugenol, citronello, limonene, butylphenyl, methylpropional, benzylsalicylate, hexyl cinnamal, linalool	-	No	Gently massage over hands
Hand sanitiser L	Isopropyl alcohol, carbomer, propylene glycol, triethanolamine, Aloe Barbadensis (Aloe Vera), leaf gel	99.90 %	No	Wet hands with products and rub briskly until dry
Hand sanitiser M	70% alcohol, leaf extract of aloe vera barbadensis, glycerin, aqua, and glutaral	99.90 %	No	apply 3 ml rub for 30 seconds
Hand sanitiser N	Alcohol denat, aqua, propylene glycol, hydroxyethylcellulose	-	No	Apply a palmful
Hand sanitiser O	Alcohol denat, Aqua, Propylene glycol, Parfum, Charmomina Recutila flower extract, Acrylates, Alkyl Acrylates, Cross Polymer, Triethanolamine	-	No	Gently massage over hands
Hand sanitiser P	Alcohol denat, Triethanolamine Benzyl, Hydroxycitroneilal, Hydrooxyisoheyl, 3 - Cyclohexene, Limonene, Linalool	-	No	Gently massage over hands
Hand sanitiser Q	Alcohol denat, Ethyl Alcohol, Glycerine, Hydrogenated castor oil, Perfume, Carbomer, Sodium Hydroxide, Hexyl Cinnamal	-	No	Apply to hands covering all surface
Hand sanitiser R	Ethyl Alcohol, Propylene glycol, Chlorohexidine gluconate, Benzaphenone-2, organic Centella, Sodium Benzanoate, Potassium Sorbate, Citric Acid, Honey Marula	-	No	Not Indicated
